# The HIV care cascade in sub‐Saharan Africa: systematic review of published criteria and definitions

**DOI:** 10.1002/jia2.25761

**Published:** 2021-07-22

**Authors:** Catrina Mugglin, Delia Kläger, Aysel Gueler, Fiona Vanobberghen, Brian Rice, Matthias Egger, International epidemiology Databases to Evaluate AIDS in Southern Africa

**Affiliations:** ^1^ Institute of Social and Preventive Medicine (ISPM) University of Bern Bern Switzerland; ^2^ Faculty of Epidemiology and Population Health London School of Hygiene and Tropical Medicine London UK; ^3^ Swiss Tropical and Public Health Institute Basel Switzerland; ^4^ University of Basel Basel Switzerland; ^5^ Centre for Infectious Disease Epidemiology and Research (CIDER) University of Cape Town Cape Town South Africa; ^6^ Population Health Sciences Bristol Medical School University of Bristol Bristol UK

**Keywords:** HIV, care cascade, sub‐Saharan Africa, ART outcomes, virological suppression

## Abstract

**Introduction:**

The HIV care cascade examines the attrition of people living with HIV from diagnosis to the use of antiretroviral therapy (ART) and suppression of viral replication. We reviewed the literature from sub‐Saharan Africa to assess the definitions used for the different steps in the HIV care cascade.

**Methods:**

We searched PubMed, Embase and CINAHL for articles published from January 2004 to December 2020. Longitudinal and cross‐sectional studies were included if they reported on at least one step of the UNAIDS 90‐90‐90 cascade or two steps of an extended 7‐step cascade. A step was clearly defined if authors reported definitions for numerator and denominator, including the description of the eligible population and methods of assessment or measurement. The review protocol has been published and registered in Prospero.

**Results and discussion:**

Overall, 3364 articles were screened, and 82 studies from 19 countries met the inclusion criteria. Most studies were from Southern (38 studies, 34 from South Africa) and East Africa (29 studies). Fifty‐eight studies (71.6%) were longitudinal, with a median follow‐up of three years. The medium number of steps covered out of 7 steps was 3 (interquartile range [IQR] 2 to 4); the median year of publication was 2015 (IQR 2013 to 2019). The number of different definitions for the numerators ranged from four definitions (for step “People living with HIV”) to 21 (step “Viral suppression”). For the denominators, it ranged from three definitions (“Diagnosed and aware of HIV status”) to 14 (“Viral suppression”). Only 12 studies assessed all three of the 90‐90‐90 steps. Most studies used longitudinal data, but denominator–denominator or denominator–numerator linkages over several steps were rare. Also, cascade data are lacking for many countries. Our review covers the academic literature but did not consider other data, such as government reports on the HIV care cascade. Also, it did not examine disengagement and reengagement in care.

**Conclusions:**

The proportions of patients retained at each step of the HIV care cascade cannot be compared between studies, countries and time periods, nor meta‐analysed, due to the many different definitions used for numerators and denominators. There is a need for standardization of methods and definitions.

## 1 INTRODUCTION

The Joint United Nations Programme on HIV/AIDS (UNAIDS) adopted the 90‐90‐90 targets in 2014 to track progress towards ending the HIV epidemic. Targets to be reached by 2020 include that 90% of people living with HIV (PLWH) are aware of their status, 90% of those diagnosed initiate antiretroviral therapy (ART) and 90% of those on ART achieve undetectable viral loads to end the HIV epidemic by 2030 [1]. The HIV care cascade examines the attrition of PLWH from diagnosis of the infection to starting ART and achieving suppression of viral replication. It is used to monitor HIV programme performance and to identify gaps and opportunities for specific interventions to improve retention and outcomes [2].

The number of studies examining the HIV care cascade has increased steeply, from no such study before 2011 to over 161 studies in 2020 (based on a PubMed search combining free‐text words “HIV”, “care” and “cascade”). These studies found that in sub‐Saharan Africa and elsewhere, significant gaps remain. For example in South Africa’s North West Province, awareness of HIV status among PLWH remained similar at around 70% in 2014 and 2016 and was considerably lower in men than in women [3]. Among HIV‐positive individuals presenting for initiation of ART in Dakar, Senegal, 16% were lost to follow‐up within one year [4]. However, studies differ concerning the definitions, methods and calculations used to construct the care cascade [5, 6, 7]. It is, therefore, difficult to compare published cascade research across regions and calendar periods.

Only a few studies have evaluated the methods used to define the HIV care cascade in low‐ and middle‐income countries or sub‐Saharan Africa [2, 5, 6, 7, 8]. To fill this gap, we performed a systematic review of studies published in the academic literature from sub‐Saharan Africa to assess the different methodological approaches used to define the steps in the HIV care cascade.

## 2 METHODS

We examined guidance on systematic reviews of observational studies [9, 10] when planning this review and report our review according to the PRISMA statement [11]. The review protocol has been published [12] and was registered in Prospero (PROSPERO registration number CRD42017055863) [13].

### 2.1 Search strategy and inclusion criteria

We searched PubMed, Embase and CINAHL for articles published in English from 1 January 2004 up to 3 December 2020, the date of database search. We used Medical Subject Headings (MeSH terms) and free‐text search. The MeSH terms for HIV and AIDS, and terms “cascade”, “continuum”, “linkage to care”, “retention in care” and “ART initiation” were cross‐referenced with terms for 62 African countries. The detailed search strategy has been published elsewhere [12]. Studies were included if they reported on at least one step of the 90‐90‐90 cascade or on at least two steps of the extended cascade. Pairs of reviewers (AG and FV, CM and DK) screened titles and abstracts. Two reviewers (CM, DK) independently screened the full text of potentially eligible studies using a standardized eligibility checklist. Disagreements were resolved through discussion.

### 2.2 Definition of steps

**Figure 1:**
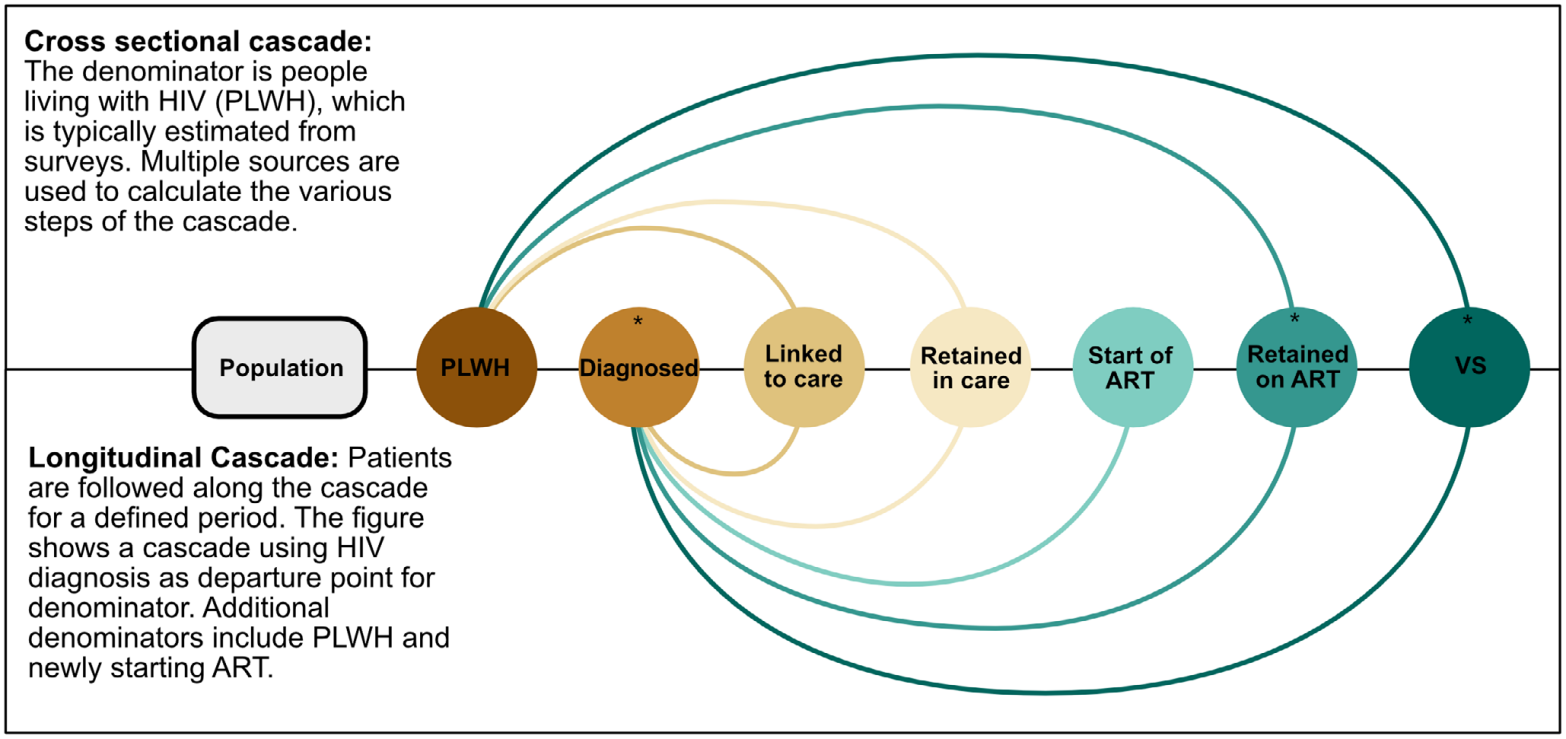
Cascade steps and denominators used for each step. The figure shows the denominators and numerators and their connection adapted from the WHO framework for cross‐sectional and longitudinal studies. *Steps of the 90‐90‐90 cascade. VS, viral suppression.

Figure 1 illustrates the populations involved in the cascade (bubbles) and the numerators and denominators (connectors linking populations) proposed by WHO and others for cross‐sectional and longitudinal cascades [6, 14, 15]. Seven populations (steps) are involved: (i) PLWH (diagnosed or undiagnosed); (ii) PLWH who have been diagnosed with HIV infection (the first 90); (iii) PLWH who have been diagnosed and linked to care; (iv) PLWH who have been diagnosed and who are retained in pre‐ART care; (v) PLWH who have been diagnosed and started ART; (vi) PLWH who have been diagnosed and are retained on ART (the second 90); (vii) PLWH who have been diagnosed, are retained on ART and are virologically suppressed (the third 90). A study reported on a step if it provided the number of patients in the numerator and denominator or a percentage of patients completing the step. We considered the step as clearly defined if the authors reported definitions for the numerator and denominator, such as the description of the population studied and methods of assessment or measurement.

### 2.3 Data extraction

We developed a standardized data extraction sheet, which was piloted by four reviewers (AG, CM, DK and ME) and revised. Data extraction was performed independently in pairs by three reviewers (CM, DK, Leona Hulbert), with disagreement resolved by discussion. Data were then entered into an EpiData database [16]. We classified study design into cross‐sectional studies, longitudinal studies and studies that combine both designs (mixed design). We defined regions of sub‐Saharan Africa according to the classification used by the International epidemiological Databases to Evaluate AIDS (IeDEA) [17].

### 2.4 Statistical analysis

Descriptive statistical analyses were performed using STATA version 14.0 (Stata Corporation, College Station, TX, USA) [18].

## 3 RESULTS

### 3.1 Selection of eligible studies

**Figure 2:**
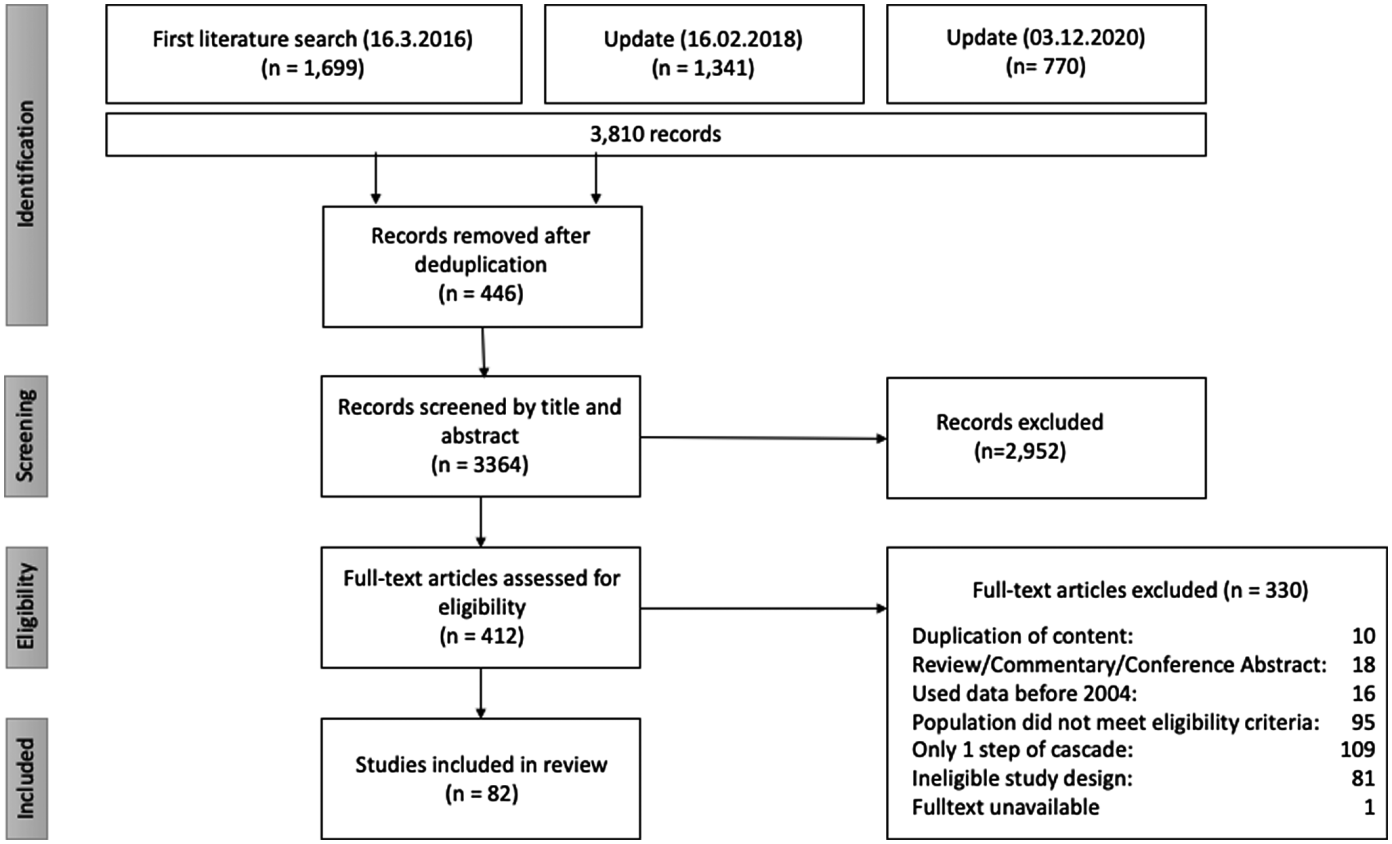
Flowchart of study selection.

Overall, 3810 articles were identified (Figure 2). After removal of duplicates, 3364 articles were screened by title and abstract, of which 412 were considered potentially eligible and underwent full‐text screening. Five studies reported on data from the Kenya AIDS Indicator Survey 2012 (KAIS) [19]. To avoid double‐counting of data from the KAIS, we included the publication reporting on the largest number of steps in the cascade [20]. A total of 82 studies met the inclusion criteria.

### 3.2 Study characteristics

The median year of publication of studies was 2015 (Table 1). The studies included a median of 1776 people; the largest study had 884,328 people and the smallest 112 people. Most studies (58 studies, 71.6%) were longitudinal, with a median follow‐up of three years. The medium number of steps covered by each study was 3 (interquartile range [IQR] 2 to 4); 12 studies assessed all three of the 90‐90‐90 steps [8, 20, 21, 22, 23, 24, 25, 26, 27, 28, 29, 30]. Most studies were from Southern Africa (38 studies, 34 from South Africa) and East Africa (29 studies). Nineteen countries were represented (Burkina Faso, Cameroon, Côte d'Ivoire, Ethiopia, Gabon, Kenya, Lesotho, Malawi, Mozambique, Nigeria, Rwanda, Senegal, Sierra Leone, South Africa, Tanzania, Togo, Uganda, Zambia and Zimbabwe).

**Table 1:** Characteristics of 82 published studies reporting on the HIV care cascade in sub‐Saharan Africa

Characteristic	
Year of publication	2015 (2013 to 2019)
No. of people included	1776 (559 to 7536)
Study design	
Longitudinal study	58 (71.6%)
Cross‐sectional study	18 (22.2%)
Mixed design^a^	5 (6.25)
No. of steps covered	3 (2 to 4)
Duration of follow‐up (years)^b^	3 (2 to 6)
Total no. of countries included	19
Region	
Central Africa	4 (4,9%)
East Africa	29 (35.4%)
Southern Africa	38 (46.3%)
West Africa	7 (8.5%)
Multi‐regional study	4 (4.9%)
No. of sites^c^	2 (1 to 15)
No. of studies at national level	2 (2.4%)
Data collection methods	
Routine clinical records	61 (74.4%)
Questionnaires	35 (42.7%)
Blood samples collected specifically for study	33 (40.2%)
Clinical records extracted from^d^	
Paper files only	6 (9.8%)
Paper and electronic files	10 (16.4%)
Electronic files only	28 (45.9%)
Not reported	17 (27.8%)

Median (interquartile range) or number (%) are shown.

^a^ Studies combining cross‐sectional and longitudinal datasets

^b^ data for the 58 longitudinal studies

^c^ studies not reporting on national level or mobile testing (N = 52)

^d^ 61 studies where clinical records were used.

### 3.3 Data collection and management

In 61 (74.4%) of the studies, data collection was performed by the clinic or hospital where participants were in care. Sixty‐one (74.4%) studies used data collected through routine clinical records, 35 (42.7%) used questionnaires and 33 (40.2%) studies collected samples specifically for the study. Twenty‐eight studies (45.9%) reported using data captured in an electronic database only, and most of the studies (56, 68.3%) had a personal identifier assigned to study participants, which allowed for tracking of the participants along the cascade (Table 1).

### 3.4 Number of definitions of numerator and denominator

The number of different definitions used in the 82 studies for the numerators defining the different steps ranged from four definitions (for step “People living with HIV”) to 21 (step “Viral suppression”). Similarly, the number of different denominators ranged from three (step “Diagnosed and aware of HIV status”) to 14 (step “Viral suppression”) (Table 2). The median number of definitions used across the seven steps was 8 for the numerator and 8 for the denominator.

**Table 2:** Reporting and definitions provided on seven HIV care cascade steps in 82 published studies

	HIV care cascade step	Description	Study design	No. of studies reporting on step (% of 82 studies)	No. of studies reporting definition for numerator and denominator (% of studies reporting on step)	No. of definitions used for numerator	No. of definitions used for denominator
						Description of definitions	Description of definitions
1	People living with HIV	The proportion of people testing positive for HIV	10 Cross‐sectional 3 Longitudinal 2 Mixed study design^b^	15 (18%)	14 (93%)	4	5
						People tested HIV positive in study through: ‐ HTC rollout ‐ Outreach campaign ‐ Home‐based testing and counselling People tested HIV positive OR self‐reporting being HIV positive	People tested for HIV: ‐ Adult residents of a high prevalence community ‐ Participants of testing campaign ‐ Participants of home‐based counselling and testing (HBCT) in a survey among resident adults ‐ Random sample of population ‐ Participants in cross‐sectional survey
2^a^	Diagnosed and aware of their status	The proportion of people living with HIV who are aware of their status	10 Cross‐sectional 10 Longitudinal 4 Mixed study design	24 (29%)	19 (79%)	5	3
						Self‐reported to be HIV positive Awareness of HIV‐positive status either through VCT at the sero‐survey or through self‐reporting. HIV infection assessed through: ‐ Clinical records ‐ Having a previous positive test ‐ Having a previous HIV related clinical visit	‐ All participants testing positive for HIV ‐ Everyone attending HIV testing ‐ Population of testing area
3	Linked to pre‐ART care	The proportion of people living with HIV linked to care	10 Cross‐sectional 15 Longitudinal 3 Mixed study design	28 (34%)	24 (86%)	5	6
						Self‐reported measures: ‐ Clinical visit ‐ Eligibility assessment for ART performed Assessed through clinical records: ‐ Recorded clinical visit ‐ Eligibility assessment for ART performed ‐ Patient engages with HIV‐related health care (clinic visit, registration in clinic, CD4 cell count, VL measurement, recorded ART start) Time point of measurement: 1 to 12 months since diagnosis	‐ All participants testing HIV positive ‐ Participants newly infected with HIV (not necessarily diagnosed) ‐ Participants newly diagnosed with HIV ‐ Participants HIV positive and aware of their status ‐ Participants reporting a positive HIV self‐test ‐ HIV‐positive participants not on ART at baseline, reporting never having initiated ART
4	Retention in pre‐ART care	The proportion of people linked to HIV care who are retained in pre‐ART care	6 Cross‐sectional 13 Longitudinal	19 (23%)	12 (63%)	8	10
						Self‐reported measures: ‐ Clinical visit ‐ Eligibility assessment completed Ascertained through clinical records: ‐ Clinical visit ‐ Eligibility assessment completed ‐ Patient engages with formal healthcare sector for HIV‐related healthcare (HIV clinic visit, registration in clinic, CD4 cell count, VL measurement, recorded ART start) Time point of measurement: ‐ 1 to 12 months since diagnosis Definitions that were not further specified but allowed for interpretation of findings: "not eligible for ART or eligible but retained in care," "retained in the HIV care cascade"	‐ Participants enrolled in study ‐ Participants newly diagnosed with HIV ‐ All participants testing HIV positive ‐ Participants reporting a positive HIV self‐test ‐ Participants HIV positive and aware of their status ‐ HIV‐positive participants not on ART at baseline ‐ HIV positive never reporting ART initiation ‐ Patients enrolled in care ‐ Participants who present for at least one follow‐up visit ‐ Participants with available CD4 cell count and VL measurements
5	ART initiation	The proportion of people linked to pre‐ART care who initiated ART	3 Cross‐sectional 32 Longitudinal 4 Mixed study design	39 (48%)	25 (64%)	9	9
						Assessed as: ‐ Self‐reported use of ART ‐ Clinical records, clinically confirmed ART initiation date ‐ Laboratory confirmation of ART in blood samples ‐ Imputed based on documented creatinine‐measurement (which is performed prior to initiation of Tenofovir) ‐ Not specified how initiation was assessed Time point of measurement: ‐ Months since eligibility criteria met (range: 3 to 12 months) ‐ Months since diagnosis (range: 1 to 12 months) ‐ At the end of study period (2.5 to 10 years) Definition that was not further specified: "Participants initiating ART"	PLWH: ‐ PLWH enrolled in the study ‐ All participants testing HIV positive and receiving their result ‐ Participants with confirmed HIV‐positive status ‐ Newly diagnosed with HIV ‐ HIV‐positive study participants willing to share their status Participants ever linked to care Enrolled in study with available VL Eligible for ART: ‐ Participants eligible for ART based on CD4 cell count or clinical staging ‐ Participants eligible for ART and linked to care ‐ Newly diagnosed with HIV and eligible for ART
6^a^	Retention on ART	The proportion of people on sustained ART who initiated ART	13 Cross‐sectional 29 Longitudinal 4 Mixed study design	46 (56%)	34 (74%)	12	8
						Self‐reported measures: ‐ Clinic visit ‐ Showing medication to study nurse ‐ ART use, including missed doses Ascertained through: ‐ Clinical records ‐ Laboratory testing (ART drug levels; “HIV related laboratory testing” not further specified) ‐ Pharmacy records/refills ‐ Pill counts Time points for measurement for longitudinal studies: ‐ On ART for: 6 month, 12 months, 2 years, 3 years ‐ At the end of the study period: 7 years, 10 years Definitions that were not further specified: "Currently on ART," “active on ART,” “self‐reported retention in care”	‐ Participants self‐reporting being HIV positive (not necessarily confirmed through a test) ‐ Participants with laboratory confirmed HIV status and reporting to have known that they are HIV positive ‐ Participants newly diagnosed with HIV ‐ Participants starting ART (self‐reported or assessed through clinical records) ‐ Participants currently receiving ART at clinics ‐ Participants that tested HIV positive (in study, random sample, cross‐sectional survey) ‐ HIV positive and available VL ‐ Participants currently in HIV care (visited a clinic within the past 6 months)
7^a^	Viral suppression	The proportion of all people receiving ART who have suppression of HIV replication	16 Cross‐sectional 32 Longitudinal 4 Mixed study design	52 (63%)	48 (92%)	21	14
						Definition of viral suppression ranged from <25 to <5000 copies/mL. Threshold was not explicitly stated in one study. Time points at which viral suppression was assessed ranged from 1 month on ART to 10 years on ART. Time point was not specified in 18 studies	‐ HIV‐positive participants who consented to a second blood sample ‐ all HIV‐positive participants ‐ newly diagnosed, linked to care, initiated ART ‐ self‐reported ART use ‐ HIV‐positive participants who self‐reported regularly accessing an ARV clinic ‐ testing ART drug positive Patients with available VL measurements ‐ at 12 months, at 10 years Patients on ART with available VL ‐ at 6 month, 12 month, 2 years, 4 years, at the end of the study period Definitions that were not further specified: “Participants initiating ART”

^a^ Correspond to UNAIDS 90‐90‐90 targets

^b^ cross‐sectional and longitudinal component.

### 3.5 Numerators and denominators along the cascade

**Figure 3:**
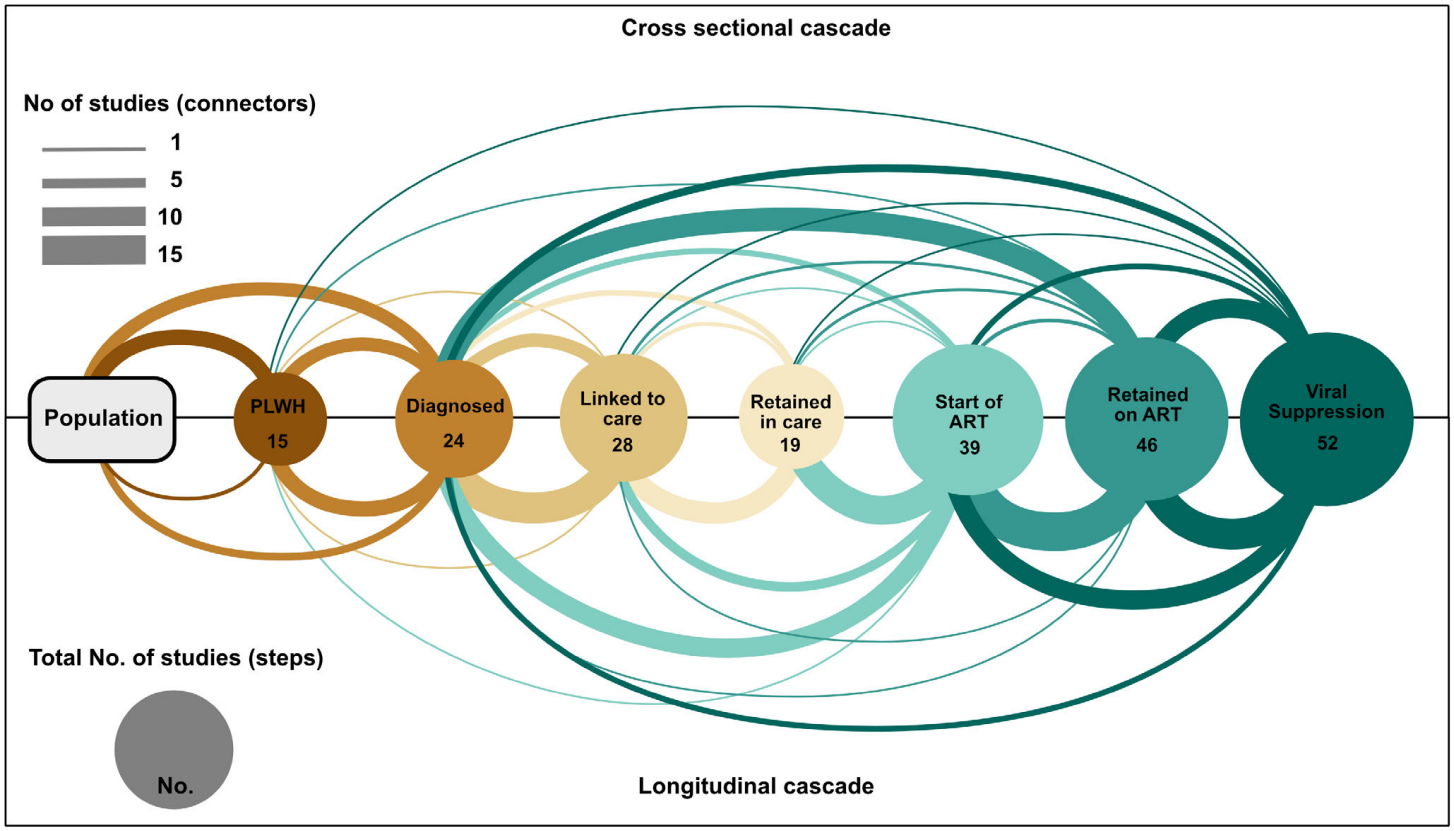
Cascade steps and denominators used for each step in 82 studies. Bubbles represent numerators, connectors point to the corresponding denominator.

The most frequently studied cascade populations were PLWH on ART with viral suppression (the third 90), followed by PLWH who have been diagnosed and are retained on ART (the second 90) and PLWH who have been diagnosed and started ART (Figure 3). In cross‐sectional studies, PLWH who had been diagnosed with HIV infection was the most common denominator used to describe retention at a subsequent step. The number of patients retained on ART was another commonly used denominator for viral suppression.

As expected, in longitudinal studies, it was common to use the previous steps as the denominator for the subsequent step (Figure 3). For example the proportion of PLWH on ART with suppression of HIV replication was based on the number who were retained on ART. The latter, in turn, was based on the number of PLWH starting ART and so forth. In recent years (2018 to 2020) the denominator used for the step “Start of ART” changed, with more studies using “Diagnosed with HIV,” rather than “Retained in care” (Figure S1).

### 3.6 Individual steps of the cascade

#### 3.6.1 People living with HIV

Fifteen studies reported numbers or percentages of PLWH [8, 22, 23, 24, 26, 27, 28, 29, 31, 32, 33, 34, 35, 36]: 10 were cross‐sectional, three were cohort studies and two of mixed design. All but one reported on the setting in which the testing took place (Table 2) and used the number who tested HIV positive as their definition of the numerator. One study included self‐reported HIV status among people who declined testing. Denominators were also consistent, with all studies using the number of people tested for HIV (Figure 3). Target populations tested differed between studies, ranging from adult residents of a high prevalence community to a random sample of the general population (Table 2).

#### 3.6.2 Diagnosed and aware of HIV infection

Twenty‐four [20, 21, 22, 23, 24, 26, 27, 28, 29, 30, 31, 33, 34, 35, 37, 38, 39, 40, 41, 42, 43, 44, 45, 46] studies reported on people diagnosed with HIV and aware of their status, with 19 providing definitions for the denominator and numerator. Ten were cross‐sectional, 10 longitudinal and 4 included longitudinal and cross‐sectional components (mixed design). Awareness of HIV infection either relied on self‐reported information (questionnaires or interviews) or was assessed through clinical records; four studies did not specify the source of information. In most studies, denominators included the PLWH testing positive for HIV, but people attending HIV testing or the whole population were also used (Table 2). Only for eight studies, HIV diagnosis was the starting point of the cascade.

#### 3.6.3 Linkage to pre‐ART care

Twenty‐eight studies reported on this step [20, 23, 24, 26, 27, 28, 31, 33, 34, 35, 37, 38, 39, 41, 44, 45, 47, 48, 49, 50, 51, 52, 53, 54, 55, 56, 57, 58]: 15 cohort studies, 10 cross‐sectional and three of mixed design. Twenty‐four studies (86%) provided a clear definition of the step (Table 2). Numerators were based on self‐reported clinic visits or assessments of eligibility for ART, or clinical records, with studies assessing linkage from 1 to 12 months since diagnosis (Table 2). Denominators were homogenous, defined as patients newly diagnosed with HIV or patients testing HIV positive (Figure 3).

#### 3.6.4 Retention in pre‐ART care

Nineteen studies reported on retention in pre‐ART care [2, 20, 26, 27, 34, 49, 50, 51, 53, 54, 55, 56, 59, 60, 61, 62, 63, 64, 65], with 12 defining numerators and denominators. Six studies were cross‐sectional and 13 longitudinal. The definitions for numerators differed for the type of information used (self‐reported clinical visit or eligibility assessment, or clinical records) and the time of assessing retention after HIV diagnosis. Denominators were heterogeneous and included people newly diagnosed with HIV, PLWH who were not eligible for ART or not receiving ART, participants who present for at least one follow‐up visit or people receiving same‐day HIV testing (Table 2).

#### 3.6.5 Initiation of ART

Thirty‐nine studies reported on initiation of ART [2, 21, 23, 28, 33, 34, 37, 40, 41, 42, 44, 45, 46, 47, 49, 50, 51, 52, 53, 54, 55, 56, 57, 58, 59, 60, 61, 62, 64, 66, 67, 68, 69, 70, 71, 72, 73, 74, 75], with 25 (64%) defining numerators and denominators. Thirty‐two were longitudinal, four of mixed design and three were cross‐sectional. Initiation of ART was either self‐reported or ascertained through clinical records. Most studies used participants eligible for ART based on CD4 cell count or clinical staging as the denominator. Three studies used all PLWH as the denominator (Table 2). The denominator and numerator also differed regarding the time point at which initiation of ART was assessed (certain time point since eligibility for ART; times since diagnosis and number of initiations at the end of the study period) (Table 2).

#### 3.6.6 Retention on ART

Overall 46 studies [2, 20, 21, 22, 23, 24, 26, 27, 28, 29, 30, 32, 40, 43, 45, 49, 50, 51, 53, 61, 63, 65, 68, 69, 72, 74, 75, 76, 77, 78, 79, 80, 81, 82, 83, 84, 85, 86, 87, 88, 89, 90, 91, 92, 93, 94] reported on retention on ART or being on ART and 34 (74%) provided clear definitions of numerators and denominators. The majority of studies were longitudinal (29 studies, 63%), 13 were cross‐sectional and four of mixed design. Studies not reporting a clear definition described this step as "currently on ART" or similar. Numerators were either self‐reported or ascertained through clinical records, pharmacy records or pill counts (Table 2). Time points for assessing numerators ranged from retention for six months to ten years of ART. The most frequently used denominator was based on the patients starting ART (26 studies, 56%) (Figure 3).

#### 3.6.7 Viral suppression

A total of 52 studies reported numbers or percentages of patients retained at this step [8, 20, 21, 22, 23, 24, 25, 26, 27, 28, 29, 30, 31, 32, 34, 36, 40, 41, 42, 43, 46, 56, 58, 65, 68, 69, 72, 73, 74, 76, 77, 78, 79, 80, 81, 82, 83, 84, 85, 86, 87, 88, 89, 90, 91, 92, 93, 95, 96, 97, 98, 99]. The majority of studies (32 studies, 62%) covering this step were longitudinal studies. Viral suppression was based on laboratory results; one of the studies used self‐reported viral suppression as the definition for the numerator. Variable definitions of viral suppression and time points when viral load was measured led to different denominators. Thresholds used for definition of viral suppression ranged from <25 to <5000 copies/mL. The threshold most frequently used was <400 copies/mL (15 studies, 31%) (Table 2). Time points at which viral suppression was assessed ranged from one month to ten years on ART and was not specified in 18 (35%) out of the 52 studies. The most frequent time point for the assessment of viral suppression was 12 months after ART initiation (15 studies, 31%). The denominators used varied widely. It ranged from all PLWH patients to patients with available viral load (VL) measurement at 10 years (Table 2).

## 4 DISCUSSION

This systematic review of published studies from sub‐Saharan Africa examined the approaches used to define the steps in the HIV care cascade and the steps covered in studies. Its results show that the definitions of the steps along the HIV care cascade are highly heterogeneous. Only 12 studies assessed all three 90‐90‐90 steps. Many studies focused on a subset of steps, with most studies assessing the start of ART, retention on ART, and achieving viral suppression. These steps are easier to assess within a typical clinic‐based cohort and are also part of the 90‐90‐90 goals proposed by UNAIDS, which might explain the numerous studies reporting on these steps. Fewer studies examined the earlier steps, including the proportion of PLWH who have been diagnosed with HIV infection (the first 90) or the step of linkage to care.

The various definitions used for numerators and denominators mean that the proportions of patients retained at each step cannot easily be compared between studies, countries and time periods. Also, the statistical combination of data in meta‐analyses, for example, for one country or region, would be problematic in this situation because "apples are combined with oranges" [100]. The heterogeneous definitions may be explained by the focus of studies, which may have been on retention in general, not specifically on the 90‐90‐90 targets. Of note, for the step of viral suppression, where definitions could be expected to be more consistent than for other steps, we identified a total of 21 different numerator definitions. Changing viral load thresholds over time and different time‐points of measuring contributed to the many different numerators, reflecting changing recommendations for defining viral suppression. The denominators also varied widely, ranging from all PLWH to patients starting ART. In recent years, PLWH diagnosed with HIV became a more common denominator for the proportion of patients on ART, reflecting the introduction of the “treat‐all” guidelines. “Retention in pre‐ART” care does no longer apply with “treat‐all”.

Previous studies from high‐income and lower‐income settings have highlighted the lack of uniformity in definitions [5, 6, 7, 101, 102]. To the best of our knowledge, this is the first comprehensive review of the methods used to assess the full HIV care cascade in sub‐Saharan Africa, including the definitions of numerators and denominators from cross‐sectional and longitudinal studies. Most previous studies focused on one part of the HIV care cascade, for example highlighting the lack of uniformity following specific community‐based testing strategies [103, 104], or on other settings, for example high‐income countries [7, 105]. Several studies from high‐income settings have previously highlighted the heterogeneity and lack of standardization [101, 102]. Haber and colleagues recommended investments in population‐based, longitudinal cohorts so that data can be linked at the individual level across steps (denominator–denominator linkage) and within steps (denominator–numerator linkage) [6]. We found that the majority of studies reporting on the cascade use longitudinal rather than cross‐sectional data. However, denominator–denominator or denominator–numerator linkages over several steps were rare. Globally agreed‐upon measures to quantify the HIV care cascade are needed to evaluate the scope of and factors that are associated with attrition along the cascade [6, 14, 105, 106].

A strength of our study is that we included smaller surveys and cohorts that are not necessarily representative of the national level but highly relevant to many settings in sub‐Saharan Africa. Some weaknesses need to be considered. Our review was based on a systematic search of the academic literature and did not consider other reports on the HIV care cascade. For example we did not include reports published by governments describing national care continua. Of note, a systematic review of national data found that only a few complete national continua of care were available, and there was much heterogeneity in the methods for determining progress towards the 90‐90‐90 target [5]. A previous systematic review identified 13 cascades from the USA, Canada, Denmark, Georgia and Australia and recommended the use of population‐based data sets to improve comparability [7]. Another study examined the linkage steps to care and ART initiation following community‐based detection of HIV [107]. Similar to our research, the authors found that definitions of numerators and denominators and observation periods were heterogeneous and that a meta‐analysis was inappropriate. We did not examine disengagement and reengagement in care. Such “churning in and out of HIV care” was beyond the scope of this review [108].

We did not systematically search for reports from the Population‐based HIV Impact Assessment (PHIA) surveys and did not include them [109, 110]. The PHIA project performed nationally representative cross‐sectional surveys for 13 different countries, measuring the three 90‐90‐90 steps of the HIV care cascade. In contrast to the academic literature, multinational studies, which use a standardized methodology, can provide data that are comparable across countries. Where a population‐based nationwide cohort is missing, combined information from cross‐sectional studies, cohort studies and from health surveillance is often used to evaluate the HIV cascade of care. A weakness that applies both to the PHIA surveys and the academic literature is the limited coverage of data on the care cascade. In our review, we identified studies from 19 countries in sub‐Saharan Africa. Therefore, data on the care cascade may still be lacking for some countries, including countries profoundly affected by the HIV epidemic. The IeDEA collaboration is an extensive network of clinical cohorts, which can address the later steps of the HIV cascade in a standardized way [17, 111].

## 5 CONCLUSIONS

In conclusion, the many different numerators and denominators used along the HIV care cascade limit the comparability between studies. It is not clear whether observed differences in results are due to real differences or different approaches to the calculation of numerators and denominators. Future studies assessing the HIV care cascade should provide clear definitions of numerators and denominators used. To identify gaps and opportunities for specific interventions to improve the cascade of care, we need standard definitions, such as those definitions proposed by the WHO [14].

## Competing interests

The authors declare no competing interests.

## Authors’ contributions

AG, BR, CM, FV and ME conceived the study and wrote the protocol. AG, FV, CM and DK performed title and abstract screening. AG, CM and DK performed full‐text screening. CM and DK performed data extraction and analysis. CM and ME wrote the first draft of the paper. All authors reviewed and approved the final manuscript.

## Supporting information


**Figure S1**. Cascade steps and denominators used for each step. Bubbles represent numerators; connectors point to the corresponding denominator. Original search (upper panel) and new studies (lower panel).
