# Supplementary material for: The HIV care cascade in sub‐Saharan Africa: systematic review of published criteria and definitions
Source: J Int AIDS Soc. 2021 Jul 22;24(7):e25761. doi: 10.1002/jia2.25761 (PMC8297382; doi:10.1002/jia2.25761)

**Supplemenatry Figure S1: Cascade steps and denominators used for each step. Bubbles represent numerators; connectors point to the corresponding denominator. Original search (upper panel) and new studies (lower panel).**

Before 2018


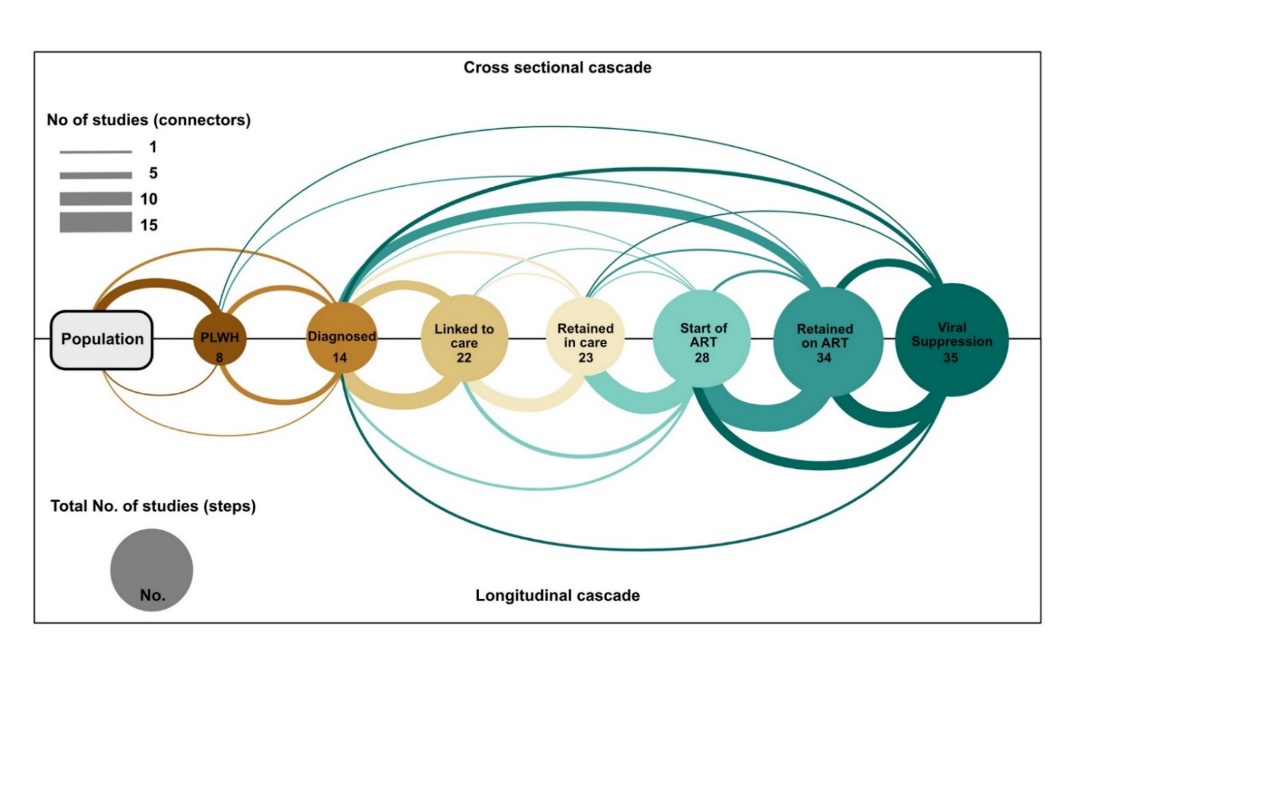


2018-2020


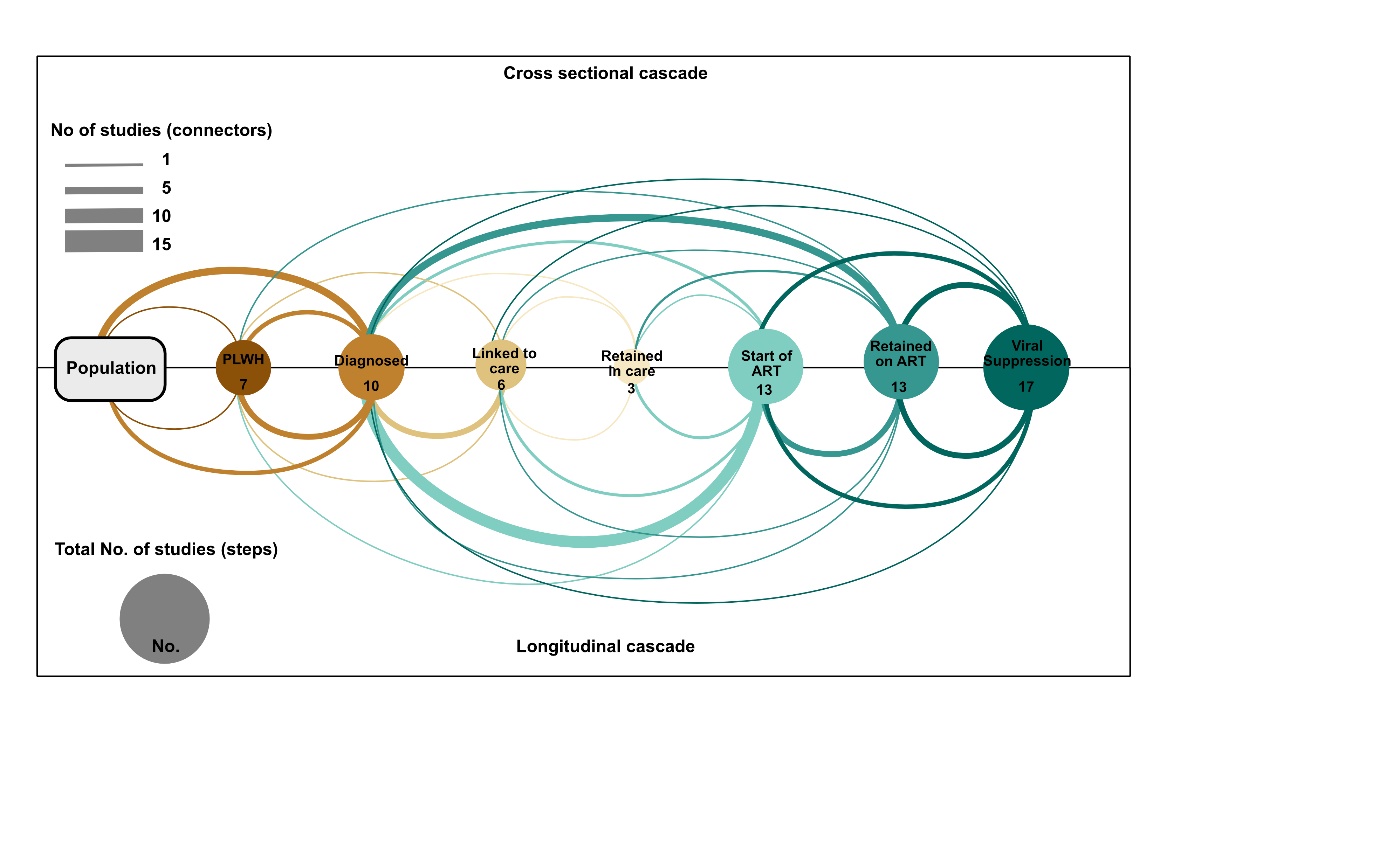

Supplement: Supplementary file 1 — Figure S1. Cascade steps and denominators used for each step. Bubbles represent numerators; connectors point to the corresponding denominator. Original search (upper panel) and new studies (lower panel). [file JIA2-24-e25761-s001.docx]
